# Simultaneous occurrence of extralobar pulmonary sequestration, esophageal duplication, and bronchogenic cysts in a Chinese child: a rare case report

**DOI:** 10.3389/fped.2024.1367626

**Published:** 2024-02-28

**Authors:** Huashan Zhao, Yunpeng Zhai, Rui Guo, Hongxiu Xu, Sai Huang, Longfei Lv, Shisong Zhang

**Affiliations:** ^1^ Department of Thoracic and Oncological Surgery, Children’s Hospital Affiliated to Shandong University, Jinan, China; ^2^ Department of Thoracic and Oncological Surgery, Jinan Children’s Hospital, Jinan, China

**Keywords:** mediastinum, extralobar pulmonary sequestration, esophageal duplication, bronchogenic cyst, Chinese children

## Abstract

The occurrence of simultaneous extralobar pulmonary sequestration, esophageal duplication, and bronchogenic cysts is relatively low. We report the case of a 9-month-old Chinese child who had a right lung cyst, detected *in utero* and was closely monitored until birth. At age 9 months, contrast-enhanced computed tomography revealed right mediastinal extralobar pulmonary sequestration and two cysts. The patient did not exhibit any abnormalities. However, the parents were concerned about the disease. Following positive psychological counseling to the parents, surgery was the strong desire. Subsequently, successful thoracoscopic surgery was performed, excising the three lesions. No postoperative complications occurred. Postoperative pathology confirmed extralobar pulmonary sequestration syndrome combined with esophageal duplication and bronchogenic cysts. The patient was followed-up at 1 and 12 months postoperatively and recovered well with no abnormal space occupation. In such cases, preoperative imaging examinations should be carefully performed, and intraoperative exploration should correspond to that before surgery to avoid lesion omission.

## 1 Introduction

### 1.1 Background

Extralobar pulmonary sequestration, esophageal duplication, and bronchogenic cysts are rarely observed in clinical practice, and all three can be managed with surgical treatment. Further, coexistence of the three conditions together is even rarer. There are cases of extralobar pulmonary sequestration complicated with bronchogenic cyst. All three have not been reported in a single case. This unique case highlights the possibility of encountering extralobar pulmonary sequestration, esophageal duplication, and bronchogenic cysts simultaneously in a single child.

## 2 Case presentation

**Figure 1:**
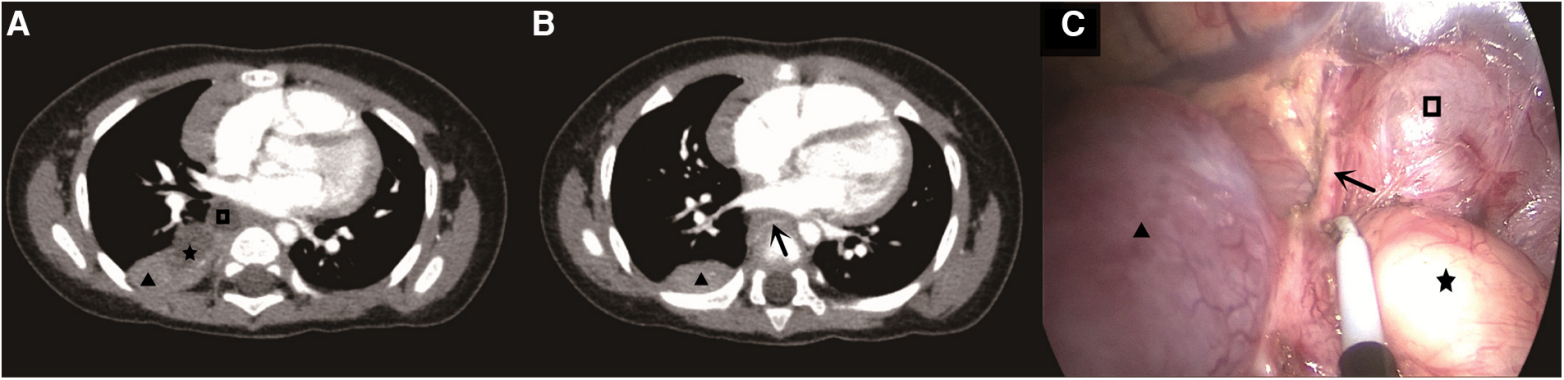
(**A**) The triangle is a extralobar pulmonary sequestration, the five-pointed star is an esophageal duplication, and the quadrilateral is a bronchogenic cyst. (**B**) The triangle is the outer extralobar pulmonary sequestration, and the arrow is the abnormal artery of the outer extralobar pulmonary sequestration. (**C**) The triangle is the outer extralobar pulmonary sequestration, the pentagram is the esophageal duplication, the quadrilateral is the bronchogenic cyst, and the arrow is the abnormal artery of the outer extralobar pulmonary sequestration.

A 9-month-old Chinese girl had been diagnosed with pulmonary sequestration in her right chest during a prenatal examination. However the fetus remained stable, and after birth, no obvious respiratory abnormalities, such as asthma or dyspnea, were observed. There was no family history of any related genetic diseases. The parents were anxious about mediastinal lesions; therefore, psychological counseling was provided. However, they strongly desired surgery. Consequently, we decided to operate on the patient. After hospitalization, the patient underwent all the relevant tests and examinations; she displayed normal development, and the chest examination was normal. Contrast-enhanced computed tomography (CT) of the patient's chest revealed an abnormal density indicative of a solid sac in the lower lobe of the right lung (Figure 1A, triangle, pentagram, quadrangle) (Figure 1B, triangle, arrow).

The presence of lesions in the mediastinum was examined preoperatively. Thoracoscopic surgery was performed under general anesthesia, during which there was red, uninflated lung tissue measuring approximately 3 × 3 × 2 cm in the right mediastinum near the hilus of the lung on the farthest side (Figure 1C, triangle). Further exploration revealed a 2 × 2 × 1 cm cystic mass (Figure 1C, pentagram) and a 1.5 × 1 × 1 cm cystic mass in the intraoperative free surrounding tissue (Figure 1C, quadrangle). During thoracoscopic surgical separation, abnormal arterial supply vessels with a diameter of approximately 0.15 cm originating from the thoracic aorta were observed (Figure 1C, arrow). We successfully resected the three lesions thoracoscopically.

On the first postoperative day, the patient resumed breastfeeding. A drainage tube inserted into the patient's chest revealed 20 ml of light-bloody fluid, but no air leakage was observed.

On the second postoperative day, the child's diet returned to preoperative levels, and 18 ml of light-bloody fluid was drained; no air leakage was observed. On the third day after surgery, the children resumed their diet. The postoperative course was uneventful.

**Figure 2:**
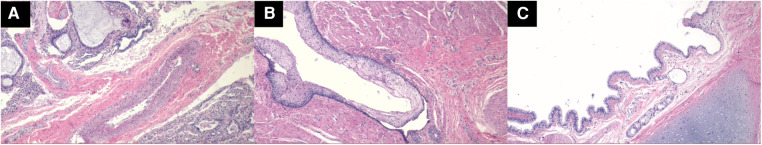
(**A**) Isolated lung HE staining magnifies pathological images by 40 times. (**B**) Pathological images of esophageal duplication magnified 40 times by HE staining. (**C**) Pathological images of bronchogenic cysts magnified 40 times by HE staining.

Pathological findings revealed foliar isolation of the lung (lung histological results; Figure 2A). Esophagogenic cysts (the squamous epithelium of the digestive tract is seen inside; Figure 2B) and bronchogenic cysts (respiratory epithelium dominated by pseudostratified ciliated columnar epithelium; Figure 2C) were also observed.

Postoperative review of children. The patient is asymptomatic with a normal CT scan.

## 3 Discussion

Pulmonary sequestration involves isolating lung tissue from the normal tracheal tree through the systemic circulation of blood. This congenital lung malformation is rare, constituting 0.15%–6.4% of all pulmonary developmental malformations (1). According to the common visceral pleural covering between the anatomical and normal lungs, there are two types of pulmonary isolation: extralobular and intralobular. Extralobar pulmonary sequestration is especially uncommon, accounting for only 15%–25% of pulmonary sequestrations (2). Approximately 60% of children with extralobar pulmonary sequestration have associated malformations, such as septal hernias, cystic adenomatoid malformations of the lungs, emphysema, and bronchogenic cysts (3). In our case, thoracoscopy revealed abnormal blood vessels in the thoracic aorta before surgery, and the solid portion exhibited extralobar pulmonary sequestration.

Esophageal duplication is a rare foregut abnormality with a very low clinical incidence (4). Bronchogenic cysts are rare congenital lung development malformations. Mampilly et al. indicated that bronchogenic cysts constitute 13%–15% of congenital intrapulmonary cyst diseases in infants. However, the pathogenesies of both esophageal duplication and bronchogenic cysts remains unclear. Esophageal duplication is often closely related to the esophagus. In our case, esophageal duplication was not closely related to the esophagus. But to those of the other two lesions. This suggests that there may be abnormalities or malformations in the development of ventral cells from the preintestinal tissues to respiratory tissues and organs during the embryonic period, possibly also leading to bronchogenic cysts. Esophageal duplication occurs when the original ventral anterior gut cells differentiate into the digestive tract tissue. This condition is rare, and our patient had pathologically confirmed esophageal duplication (5, 6). Preoperative and intraoperative diagnoses and differentiation are challenging, and postoperative pathological diagnosis is typically relied on. The duplicated esophageal wall is lined with digestive tract epithelium dominated by squamous epithelium (7), while bronchogenic cysts' inner walls consist of respiratory epithelium, characterized by pseudostratified ciliated columnar epithelium (8). In our case, although preoperative chest CT revealed two mediastinal cysts, a definitive diagnosis was not possible using preoperative imaging. Unfortunately, magnetic resonance imaging (MRI) was not performed before surgery as the parents refused the examination due to cost concerns. MRI is helpful in the diagnosis of esophageal duplication and bronchogenic cysts. However postoperative pathology confirmed the diagnosis.

Surgery is currently indicated for the treatment of exfoliar pulmonary sequestration (9, 10), esophageal duplication (11, 12), and bronchogenic cysts (13–16) in children presenting with clinical symptoms. In contrast, surgical treatment for asymptomatic cases remains controversial. Simultaneous occurrence of lobar lung isolation, esophageal duplication, and bronchogenic cysts in a single patient is exceedingly rare, and no treatment approach has been reported. In the present case, no clinical symptoms were observed. We consider the possibility of complications in particular. Thoracoscopic surgery was performed after full communication with the parents. The patient recovered well postoperatively. The operation relieved the parents' concerns and the mediastinal occupying effect on the child and was very valuable. The patient recovered well postoperatively. The operation relieved the parents' concerns. The mediastinal occupying effect on the child was relieved as well. The parents were satisfied with the surgical plan and treatment results. Thoracoscopic treatment was mainly for lobar lung isolation. We believe that the placement and positioning of the piercer is not special. Open surgery can locate the lesion in the intercostal area, similar to thoracoscopic treatment. But in this case, the advantage of thoracoscopic surgery is greater. The results were mostly good. Potential complications may include bleeding, infection, trachea injury, esophagus injury, residual cyst wall, pneumothorax, etc.

In our case, a 9-month-old Chinese child displayed extralobar pulmonary sequestration in the right mediastinum combined with an esophageal duplication and bronchogenic cyst; the three lesions were closely aligned but without duct communication. This unique case highlights the importance of raising clinical awareness among medical professionals and emphasizes the need for intraoperative exploration should to align with preoperative imaging examinations to prevent lesion omission.

## Data Availability

The original contributions presented in the study are included in the article/Supplementary Material, further inquiries can be directed to the corresponding author.
